# Supplementary material for: Supplementation with Complex Phytonutrients Enhances Rumen Barrier Function and Growth Performance of Lambs by Regulating Rumen Microbiome and Metabolome
Source: Animals (Basel). 2025 Jan 16;15(2):228. doi: 10.3390/ani15020228 (PMC11758348; doi:10.3390/ani15020228)
Supplement: Supplementary file 1 [file animals-15-00228-s001.zip › animals-3398044-supplementary Tables S1-S4.pdf]

Table S1. Information on primer sequences.

Gene[1]	Nucleotide Sequences 5 0 -3 0	Size/bp	GenBank No.
ZO-1	F: TTGTAGAATCCGATGTGGG<br>R: CCTGCTGTCTTAGGAAGTGTAT	251	XM_042235170
Occludin	F: GGTAACTTGGAGACGCTT<br>R: CTGCTTGTAGGCTCTTGTAT	232	XM_015101255
Claudin-1	F: GCTTCATCCTGGCGTTTC<br>R: TCCACAGCCCCTCGTAGA	126	NM_001185016
Claudin-4	F: CTTCATCGGCAGCAACAT<br>R: ACAACAGCACGCCAAACA	191	NM_001185017
Fas	F: CCAGAGGCATACAGCATCATC<br>R: CATAGGTGTCTTCCCATTCCA	143	NM_001123003
Apaf-1	F: TGGCAGTGGTGGCTTTGT<br>R: ATCACACAATGGACCCAACTTA	106	XM_042247106
Cyt-C	F: CTACCTCCGACTCACCGACA<br>R: AGGGGAATCTGCTGACCATC	183	XM_042240814
Caspase-3	F: TGAGATGCTGAAAAAGTACGCT<br>R: CAGAATCGGTGGAAAAGGAC	104	XM_015104559
Caspase-7	F: GACAGAAGAACAGGAATGGGTG<br>R: TGGCACAAGAGCAGTCGTTA	118	XM_012102956
Caspase-8	F: CTCGGGGATACTGTTTGA<br>R: GCAGTCTTTGGTTTTGTGG	233	XM_012142477
Caspase-9	F: AGTTGGACTCGGGTTTTC<br>R: GTCTGTCTGTTGGCATTTCT	179	XM_012187488
Bax	F: CTCACTGCCTCACTCACC<br>R: AGACCACTCCTCCCTACC	173	XM_027978592
Bcl-2	F: TTTGATTTCTCCTGGCTGTC<br>R: CTGCTTTCACGAACCTTTTG	142	XM_027960877

[1]*ZO-1* = zonula occludens-1; *Apaf-1* = Apoptotic protease activating factor-1; *Cyt-C* = cytochrome c; *Bax* = B-cell lymphoma-2 associated X protein; *Bcl-2* = B-cell lymphoma-2.

Table S2. Effects of CP5 supplementation on the ruminal microbiota of lambs (phylum level) %.

Items	CON	CP5	*p*-Value
Bacteroidota	0.3938±0.0338	0.4349±0.0320	0.3938
Proteobacteria	0.2618±0.0580	0.1192±0.0339	0.0492
Firmicutes	0.2074±0.0271	0.3121±0.0280	0.0189
Spirochaetota	0.0296±0.0183	0.0319±0.0111	0.9008
Fibrobacterota	0.0104±0.0070	0.0205±0.0025	0.1906
unidentified_Bacteria	0.0172±0.0032	0.0131±0.0022	0.2968
Actinobacteriota	0.0108±0.0014	0.0153±0.0014	0.0397
Euryarchaeota	0.0004±0.0002	0.0019±0.0010	0.1765
Desulfobacterota	0.0017±0.0007	0.0049±0.0005	0.0026
Cyanobacteria	0.0009±0.0003	0.0016±0.0003	0.1131
Acidobacteriota	0.0023±0.0001	0.0005±0.0001	0.0000
Chloroflexi	0.0014±0.0001	0.0002±0.0000	0.0002
Synergistota	0.0004±0.0001	0.0012±0.0002	0.0034
Gemmatimonadota	0.0011±0.0001	0.0000±0.0000	0.0002
Verrucomicrobiota	0.0010±0.0001	0.0004±0.0001	0.0005
Gracilibacteria	0.0001±0.0001	0.0002±0.0002	0.5323
Myxococcota	0.0006±0.0000	0.0000±0.0000	0.0001
Gemmatimonadetes	0.0004±0.0001	0.0002±0.0000	0.0151
Campilobacterota	0.0001±0.0000	0.0002±0.0001	0.1254
Nitrospirota	0.0003±0.0000	0.0000±0.0000	0.0004

Table S3. Effects of CP supplementation on the ruminal microbiota of lambs (genus level) %.

Items	CON	CP5	*p*-Value
Prevotella	0.2430±0.0423	0.2048±0.0393	0.5854
Succinivibrio	0.1024±0.0419	0.0433±0.0130	0.1874
Succinivibrionaceae_UCG-001	0.1123±0.0393	0.0435±0.0129	0.1041
Rikenellaceae_RC9_gut_group	0.0452±0.0116	0.0894±0.0212	0.0723
Treponema	0.0293±0.0183	0.0278±0.0108	0.9408
Lachnospiraceae_NK3A20_group	0.0069±0.0012	0.0271±0.0113	0.0828
Prevotellaceae_UCG-001	0.0380±0.0131	0.0389±0.0099	0.9477
Selenomonas	0.0000±0.0000	0.0153±0.0126	0.2420
Succiniclasticum	0.0341±0.0103	0.0373±0.0084	0.8337
Ruminococcus	0.0071±0.0017	0.0223±0.0082	0.0744
Lactobacillus	0.0116±0.0097	0.0021±0.0006	0.3889
Dialister	0.0166±0.0020	0.0183±0.0058	0.8083
Fibrobacter	0.0104±0.0070	0.0205±0.0025	0.1848
Sharpea	0.0180±0.0062	0.0057±0.0023	0.0697
Megasphaera	0.0031±0.0015	0.0103±0.0042	0.1076
Escherichia-Shigella	0.0053±0.0034	0.0003±0.0001	0.1457
Olsenella	0.0058±0.0012	0.0124±0.0012	0.0020
Shuttleworthia	0.0054±0.0020	0.0038±0.0003	0.5140
Acidaminococcus	0.0040±0.0009	0.0080±0.0019	0.0610
Acetitomaculum	0.0022±0.0003	0.0068±0.0018	0.0202

Table S4. Metabolomic analysis of differentially expressed metabolites in rumen contents

Items	FC	log2FC	VIP	*p*-Value	Type
All-Trans-13,14- Dihydroretinol	11.98	3.58	3.10	0.000	up
Ursolic acid	9.32	3.22	2.86	0.000	up
Chenodeoxycholic acid-3-beta-D-glucuronide 7-Methylguanosine	0.61	-0.71	2.01	0.001	down
Dihydroretinol	0.48	-1.07	2.00	0.001	down
bicyclo[2.2.2]oct-2-en-1-yl 4-methylbenzene-1-sulfonate	2.28	1.19	2.71	0.003	up
lithocholic acid	0.55	-0.87	1.02	0.004	down
Methionine sulfoxide	1.88	0.91	2.04	0.006	up
Isorhamnetin	2.08	1.06	1.89	0.010	up
Naringeninchalcone	0.46	-1.13	1.05	0.010	down
(1E,4E)-1,5-bis(4-methoxyphenyl)penta-1,4-dien-3-one	0.55	-0.86	1.86	0.012	down
L-Canavanine	0.56	-0.83	1.18	0.017	down
9-Oxo-10(E),12(E)-octadecadienoic acid	0.54	-0.88	1.79	0.018	down
12-Epileukotriene B4	5.27	2.40	1.69	0.020	up
Palmitoleic Acid	0.63	-0.66	1.88	0.020	down
Prostaglandin K2	0.53	-0.93	1.67	0.020	down
13(S)-HOTrE	0.61	-0.71	1.80	0.021	down
2-acetamido-3-(4-methoxyphenyl)propanoic acid	3.58	1.84	1.97	0.021	up
2,4-Dihydroxybenzoic acid	9.29	3.22	1.66	0.022	up
LPE O-17:2	2.88	1.53	1.30	0.025	up
2-{[2-(4-methylpiperazino)phenyl]methylene}hydrazine-1-carbothioamide	1.93	0.95	1.53	0.027	up
MGDG O-8:0_22:4	0.43	-1.21	1.87	0.028	down
2-Amino-1,3,4-octadecanetriol	1.89	0.92	1.72	0.033	up
Phenylacetylglutamine	1.68	0.75	1.60	0.034	up
DG O-16:3_28:7	1.98	0.99	2.30	0.034	up
Indoxylsulfuric acid	0.52	-0.95	1.41	0.039	down
N'2-benzylidene-5-hex-1-ynylfuran-2-carbohydrazide	0.44	-1.18	1.41	0.040	down
Maslinic acid	0.56	-0.84	1.58	0.042	down
Lysope 16:0	7.76	2.96	1.47	0.042	up
LPE O-16:2	7.76	2.96	1.47	0.042	up
3-Methoxy prostaglandin F1α	0.22	-2.22	1.79	0.043	down
18-β-Glycyrrhetinic acid	0.48	-1.07	2.10	0.043	down
Pilocarpine	1.56	0.64	1.47	0.045	up
2-Amino-1,3-octadecanediol	1.76	0.81	1.77	0.048	up
methyl 7-hydroxy-4-oxo-8-propyl-4H-1-benzothiine-2-carboxylate	0.51	-0.97	1.41	0.048	down
