# Supplementary material for: Supplementation with Complex Phytonutrients Enhances Rumen Barrier Function and Growth Performance of Lambs by Regulating Rumen Microbiome and Metabolome
Source: Animals (Basel). 2025 Jan 16;15(2):228. doi: 10.3390/ani15020228 (PMC11758348; doi:10.3390/ani15020228)

1. Western blot bands of *Bax*

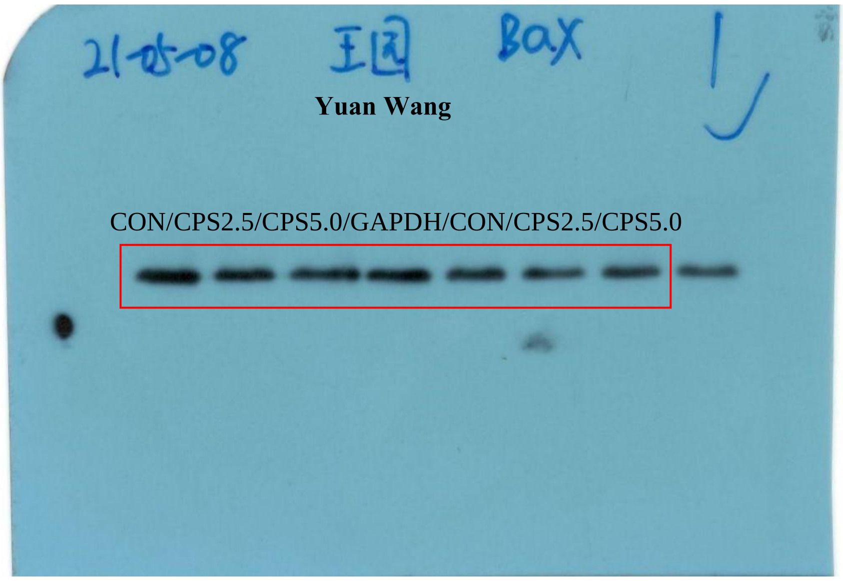

Items	CON	CPS2.5	CPS5.0	CON	CPS2.5	CPS5.0
Intensity ratio	0.76	0.37	0.38	0.75	0.34	0.37

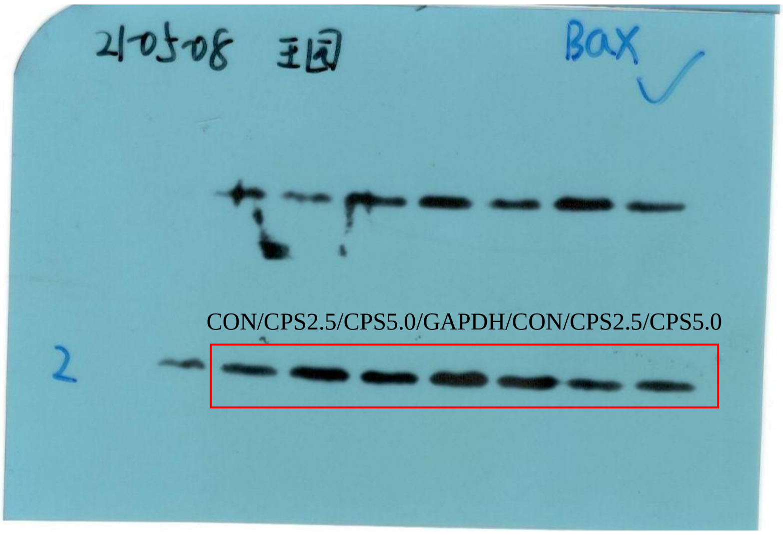

Items	CON	CPS2.5	CPS5.0	CON	CPS2.5	CPS5.0
Intensity ratio	0.47	0.50	0.54	0.53	0.35	0.32

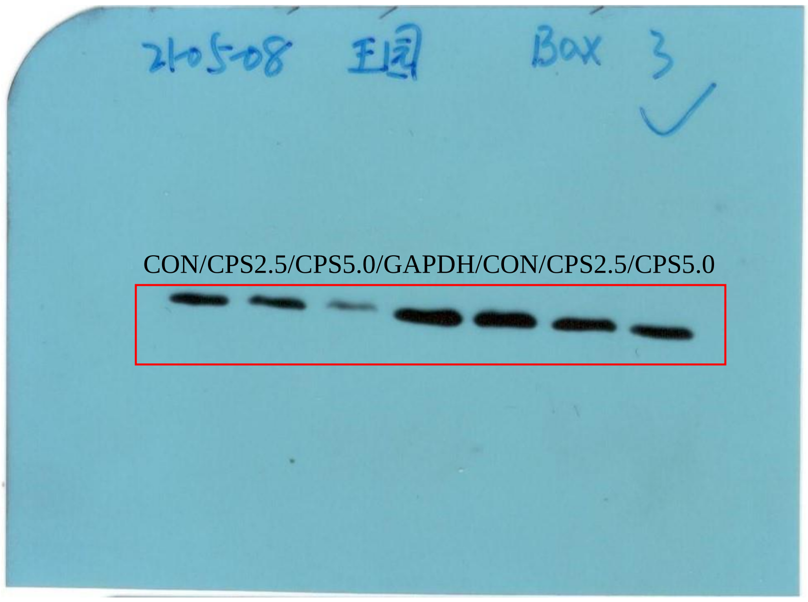

Items	CON	CPS2.5	CPS5.0	CON	CPS2.5	CPS5.0
Intensity ratio	0.53	0.31	0.24	0.73	0.38	0.35

2. Western blot bands of *Bcl2*

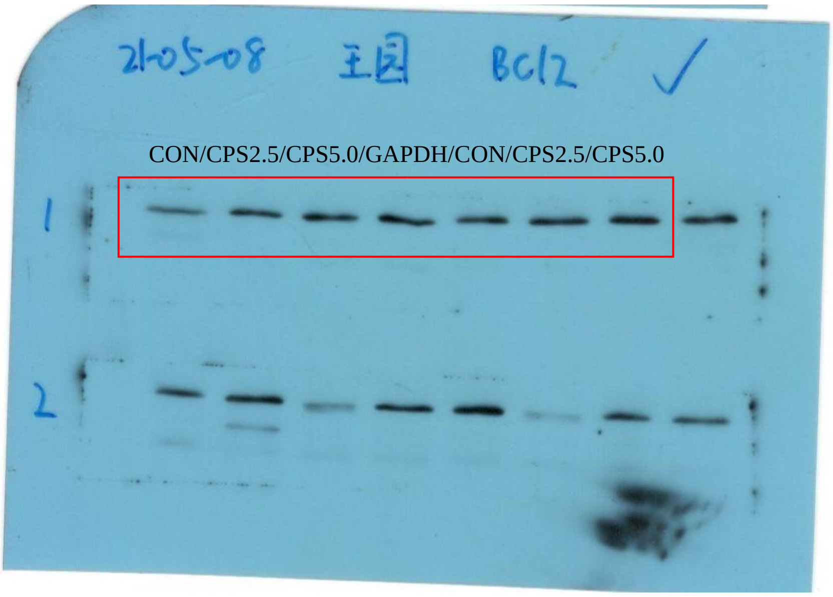

Items	CON	CPS2.5	CPS5.0	CON	CPS2.5	CPS5.0
Intensity ratio	0.10	0.28	0.52	0.19	0.23	0.56

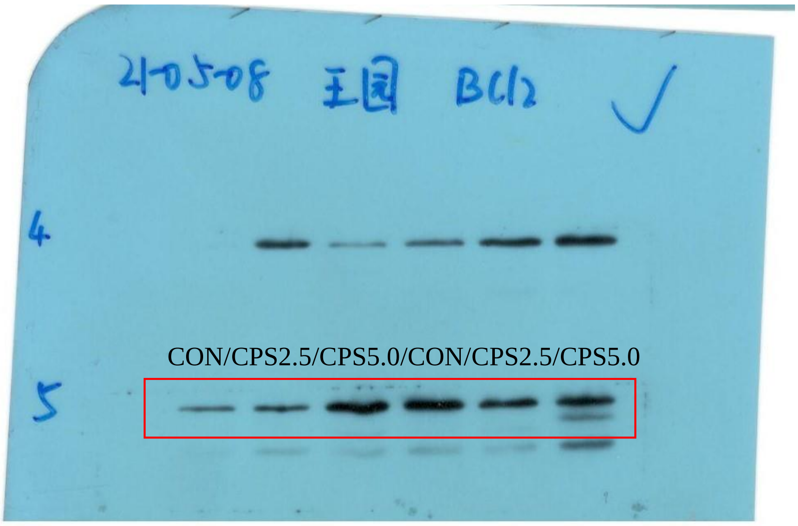

Items	CON	CPS2.5	CPS5.0	CON	CPS2.5	CPS5.0
Intensity ratio	0.16	0.30	0.78	0.64	0.48	0.56

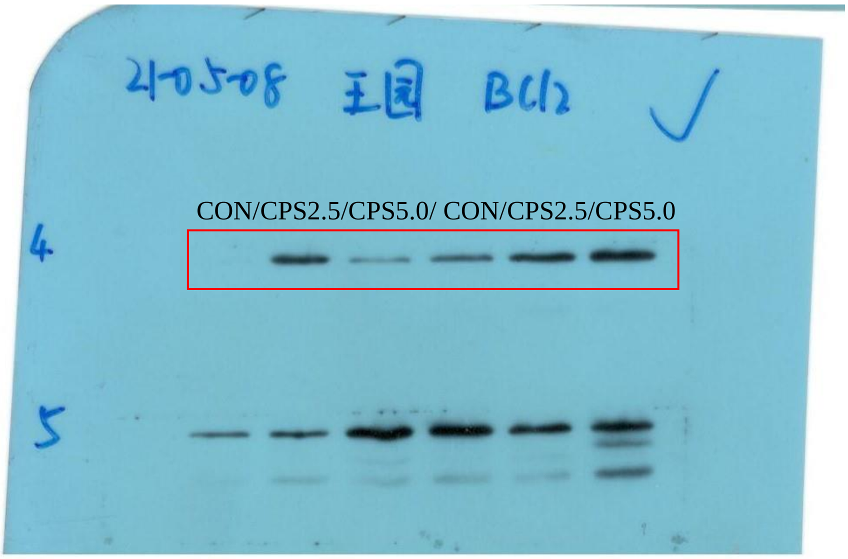

Items	CON	CPS2.5	CPS5.0	CON	CPS2.5	CPS5.0
Intensity ratio	-	0.31	0.20	0.22	0.34	0.68

3. Western blot bands of *P65*

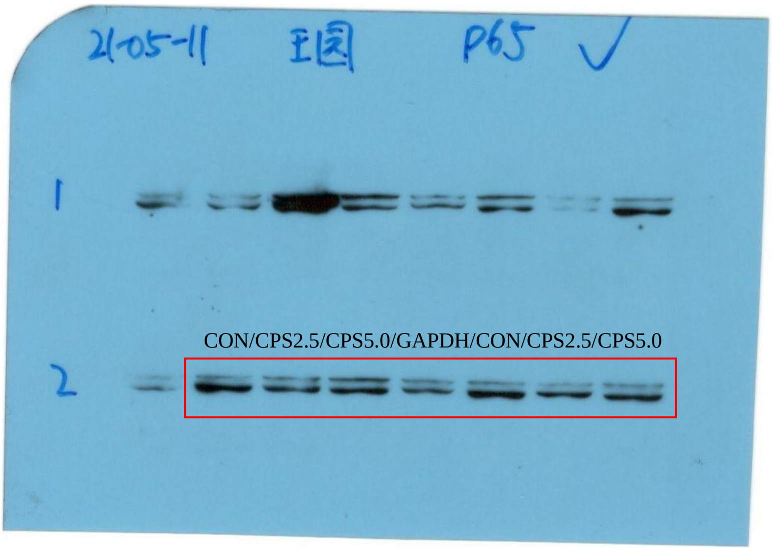

Items	CON	CPS2.5	CPS5.0	CON	CPS2.5	CPS5.0
Intensity ratio	0.58	0.41	0.28	0.65	0.40	0.29

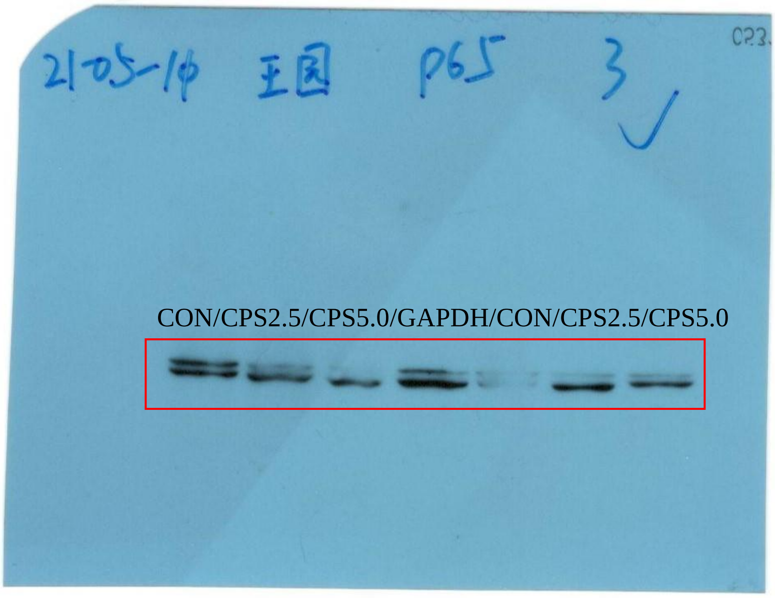

Items	CON	CPS2.5	CPS5.0	CON	CPS2.5	CPS5.0
Intensity ratio	0.51	0.25	0.24	-	0.30	0.25

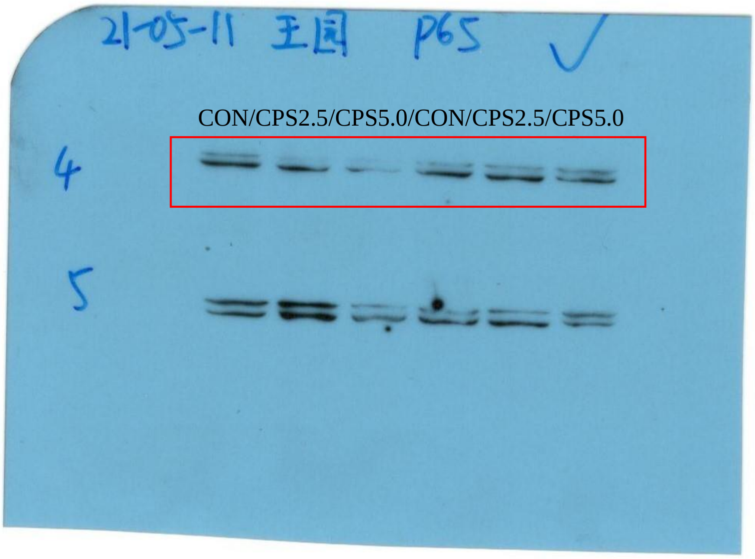

Items	CON	CPS2.5	CPS5.0	CON	CPS2.5	CPS5.0
Intensity ratio	0.42	0.31	0.19	0.33	0.29	0.23

4. Western blot bands of *caspase3*

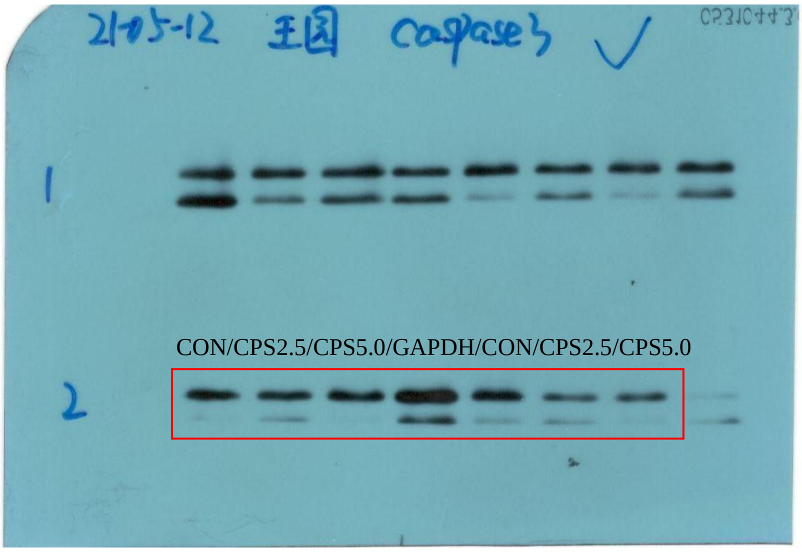

Items	CON	CPS2.5	CPS5.0	CON	CPS2.5	CPS5.0
Intensity ratio	0.53	0.31	0.35	0.56	0.26	0.27

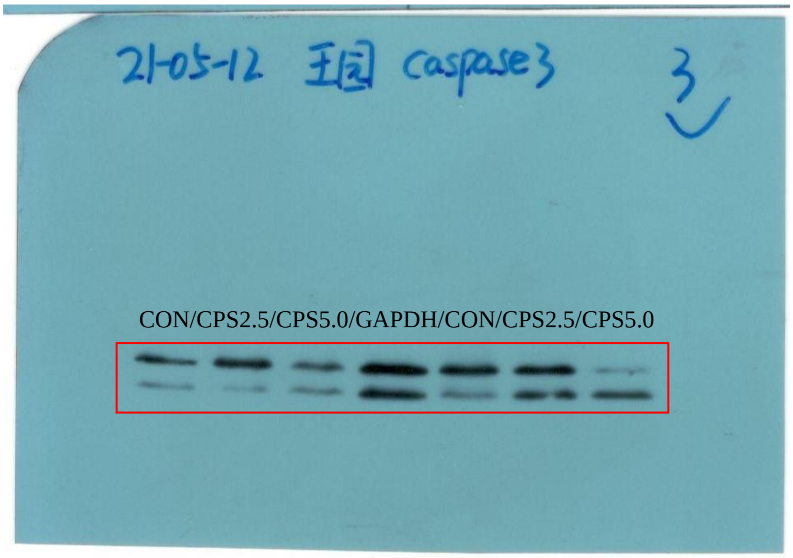

Items	CON	CPS2.5	CPS5.0	CON	CPS2.5	CPS5.0
Intensity ratio	0.30	0.49	0.31	0.52	0.55	0.24

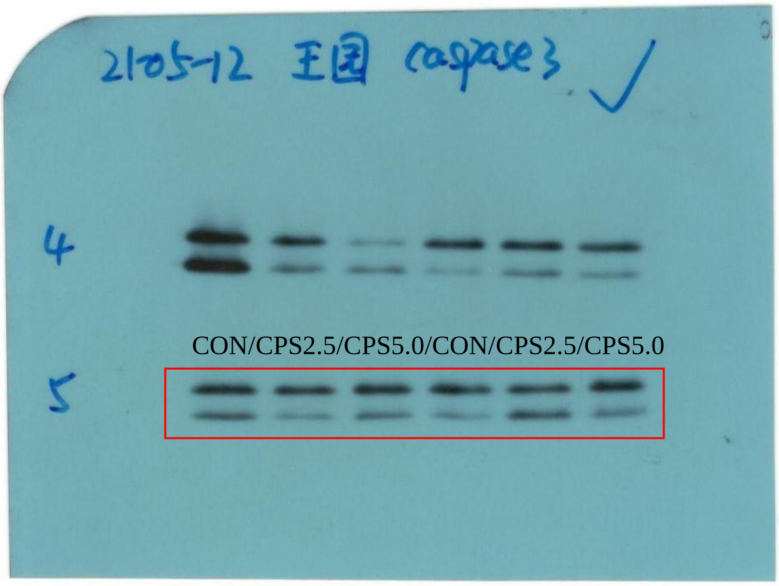

Items	CON	CPS2.5	CPS5.0	CON	CPS2.5	CPS5.0
Intensity ratio	0.52	0.32	0.31	0.47	0.30	0.31

5. Western blot bands of *JNK*

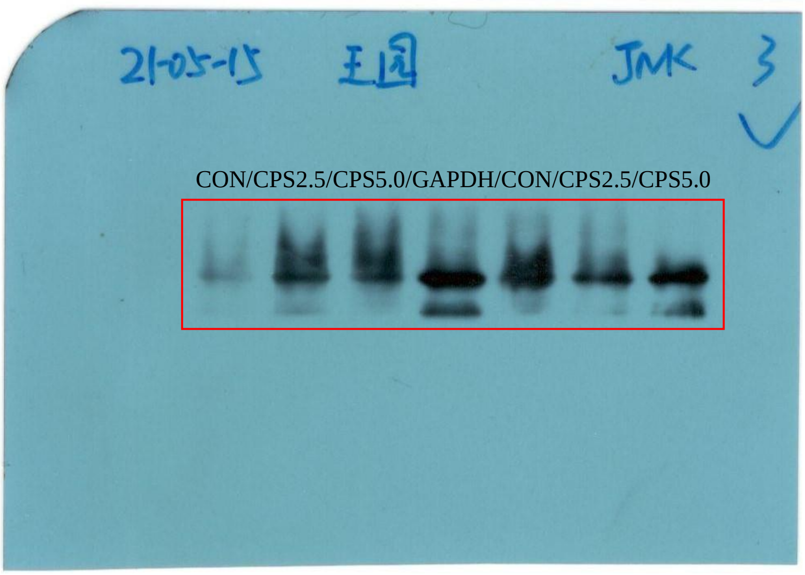

Items	CON	CPS2.5	CPS5.0	CON	CPS2.5	CPS5.0
Intensity ratio	-	0.22	0.23	0.65	0.27	0.40

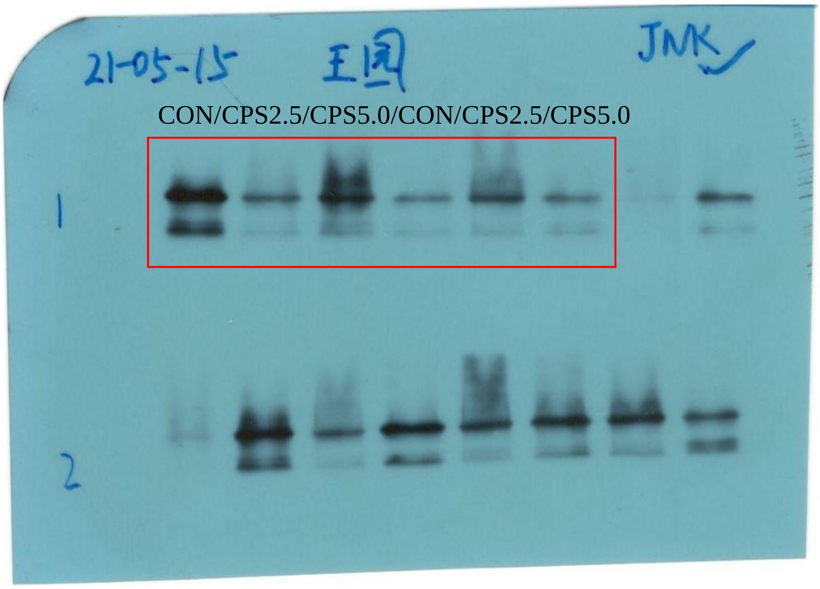

Items	CON	CPS2.5	CPS5.0	CON	CPS2.5	CPS5.0
Intensity ratio	0.57	0.13	0.31	0.18	0.27	0.16

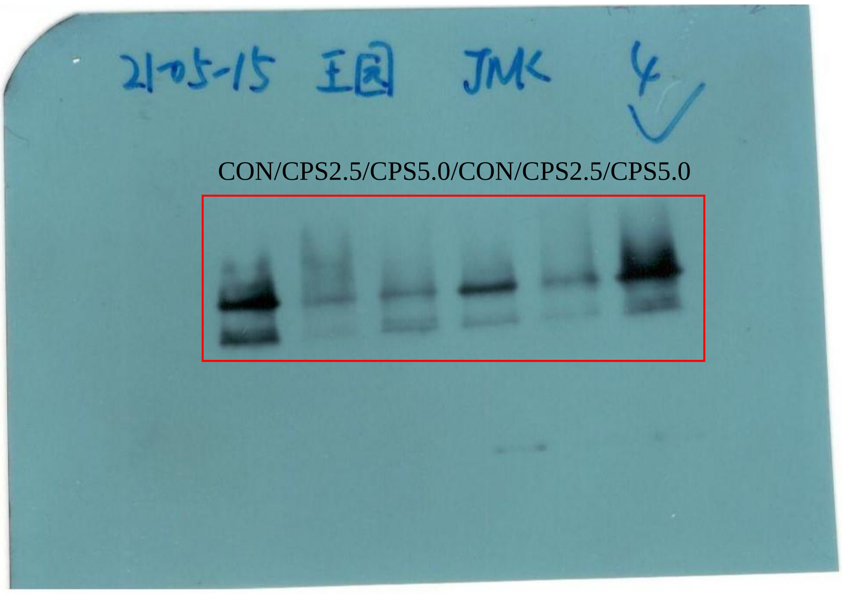

Items	CON	CPS2.5	CPS5.0	CON	CPS2.5	CPS5.0
Intensity ratio	0.50	0.17	0.18	0.32	0.18	0.45

6. Western blot bands of *P-JNK*

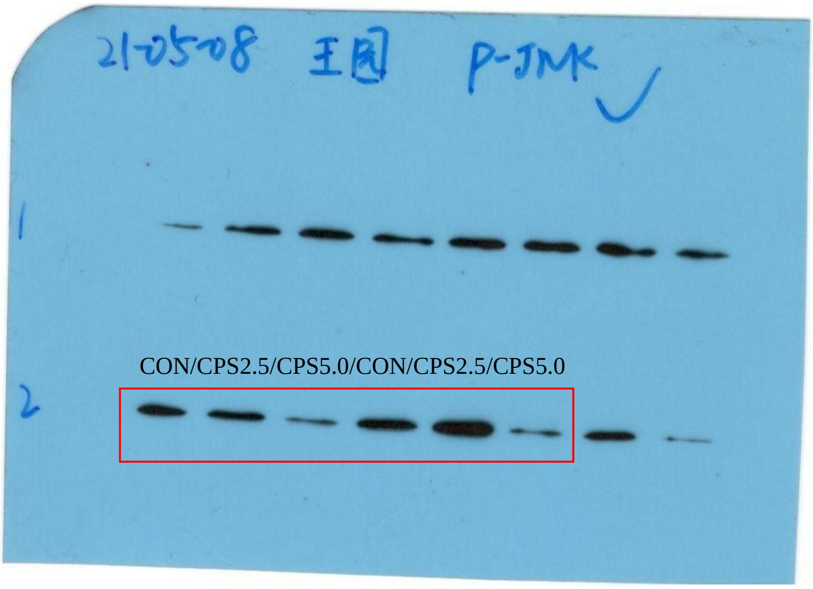

Items	CON	CPS2.5	CPS5.0	CON	CPS2.5	CPS5.0
Intensity ratio	0.63	0.49	0.36	0.65	0.70	0.28

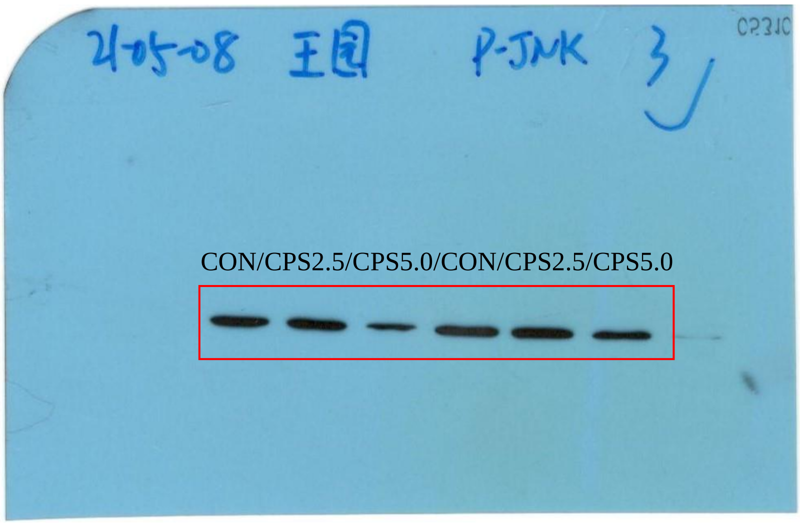

Items	CON	CPS2.5	CPS5.0	CON	CPS2.5	CPS5.0
Intensity ratio	0.62	0.60	0.28	0.60	0.58	0.42

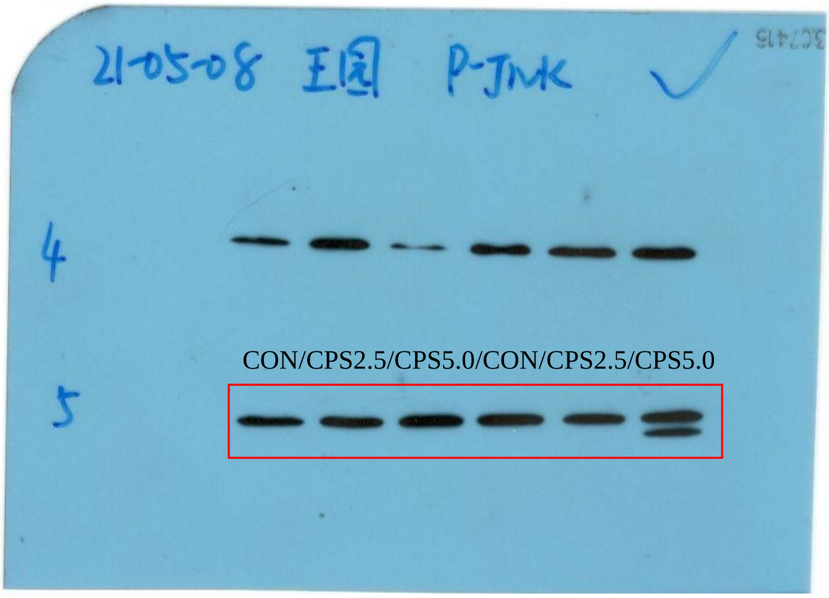

Items	CON	CPS2.5	CPS5.0	CON	CPS2.5	CPS5.0
Intensity ratio	0.60	0.56	0.58	0.59	0.49	0.50

7. Western blot bands of *P38*

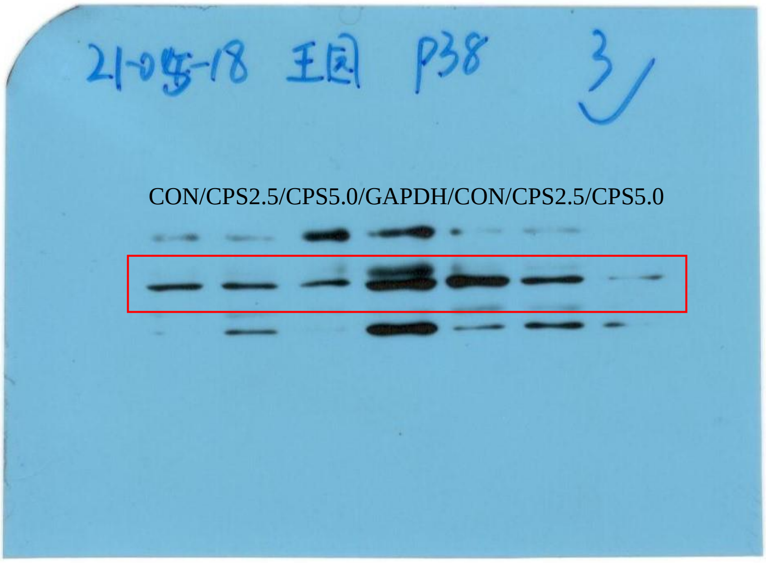

Items	CON	CPS2.5	CPS5.0	CON	CPS2.5	CPS5.0
Intensity ratio	0.31	0.30	0.20	0.40	0.36	0.07

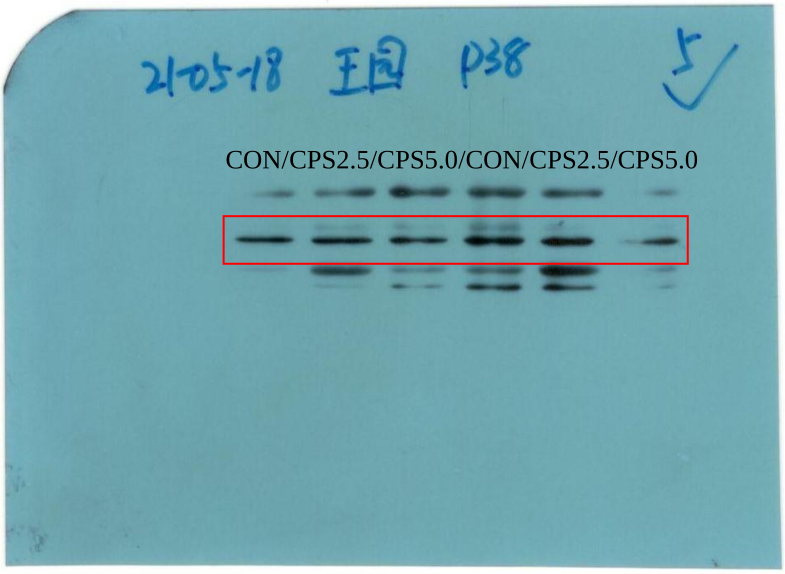

Items	CON	CPS2.5	CPS5.0	CON	CPS2.5	CPS5.0
Intensity ratio	0.28	0.27	0.18	0.43	0.33	0.09

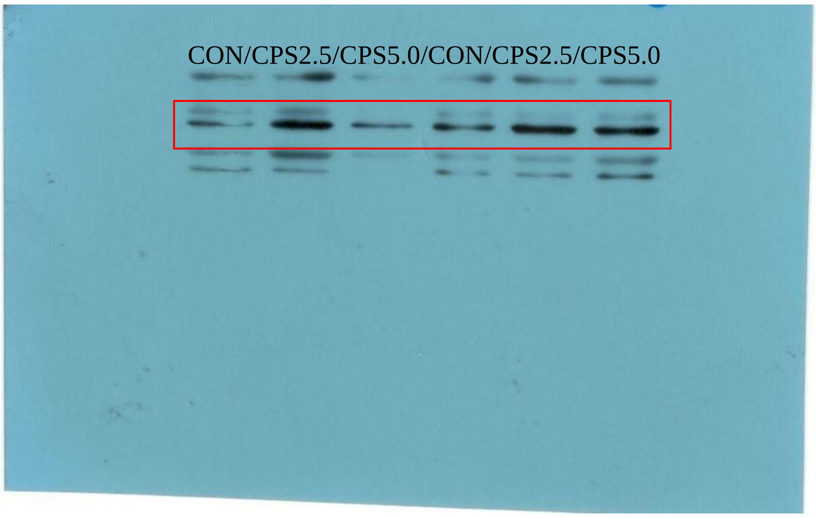

Items	CON	CPS2.5	CPS5.0	CON	CPS2.5	CPS5.0
Intensity ratio	0.32	0.33	0.25	0.18	0.33	0.12

8. Western blot bands of *P-P38*

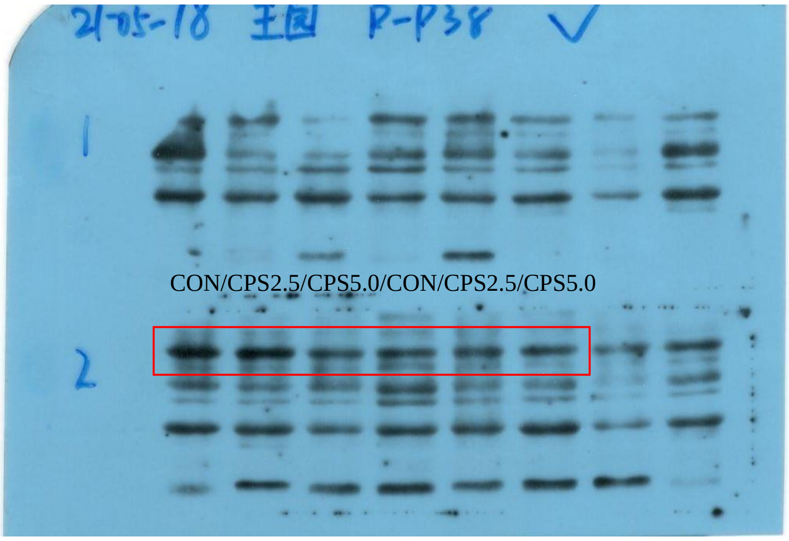

Items	CON	CPS2.5	CPS5.0	CON	CPS2.5	CPS5.0
Intensity ratio	0.45	0.43	0.25	0.34	0.28	0.27

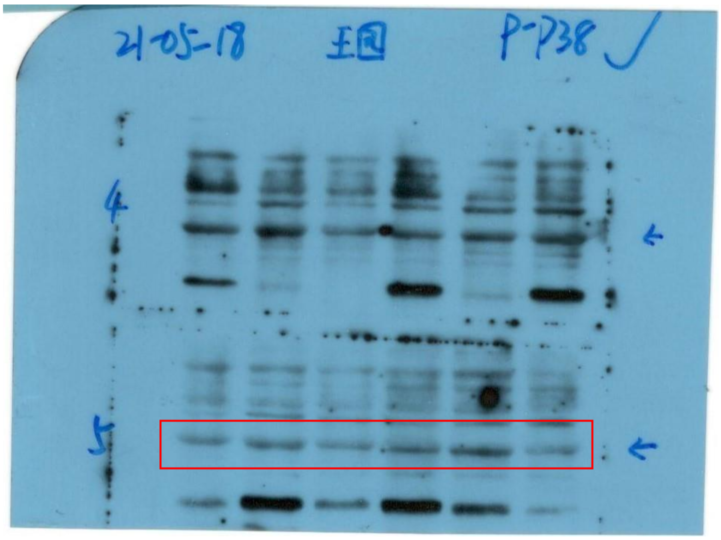

CON/CPS2.5/CPS5.0/CON/CPS2.5/CPS5.0

Items	CON	CPS2.5	CPS5.0	CON	CPS2.5	CPS5.0
Intensity ratio	0.33	0.31	0.21	0.28	0.36	0.19

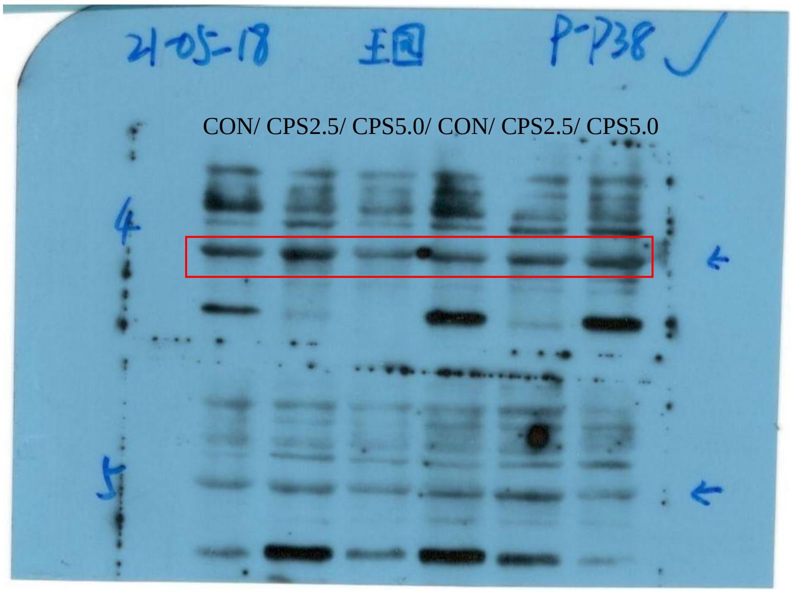

Items	CON	CPS2.5	CPS5.0	CON	CPS2.5	CPS5.0
Intensity ratio	0.37	0.40	0.22	0.32	0.35	0.33

9. Western blot bands of *GAPDH*

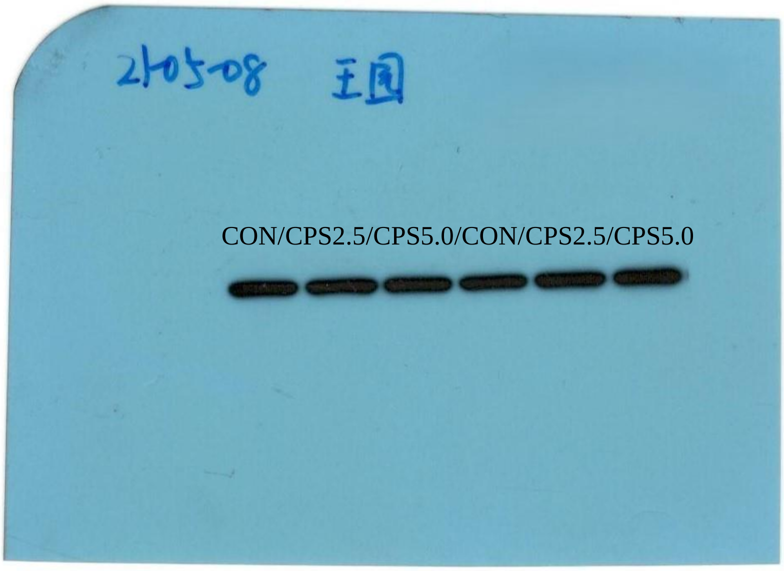

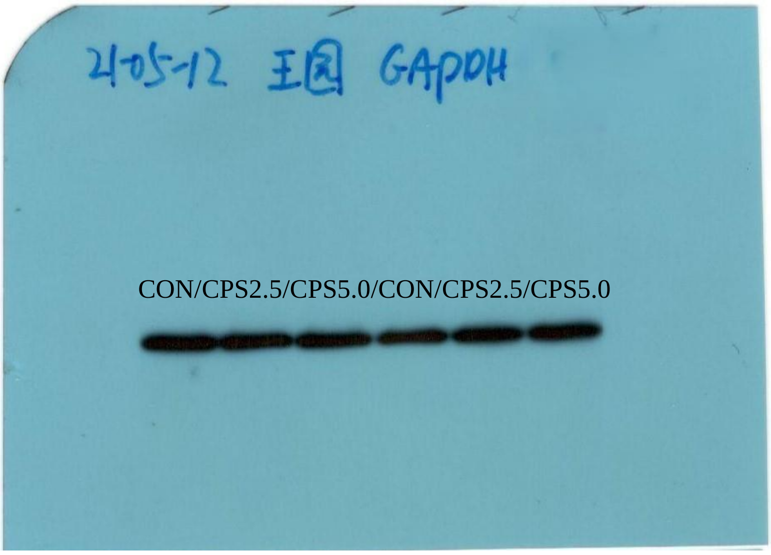

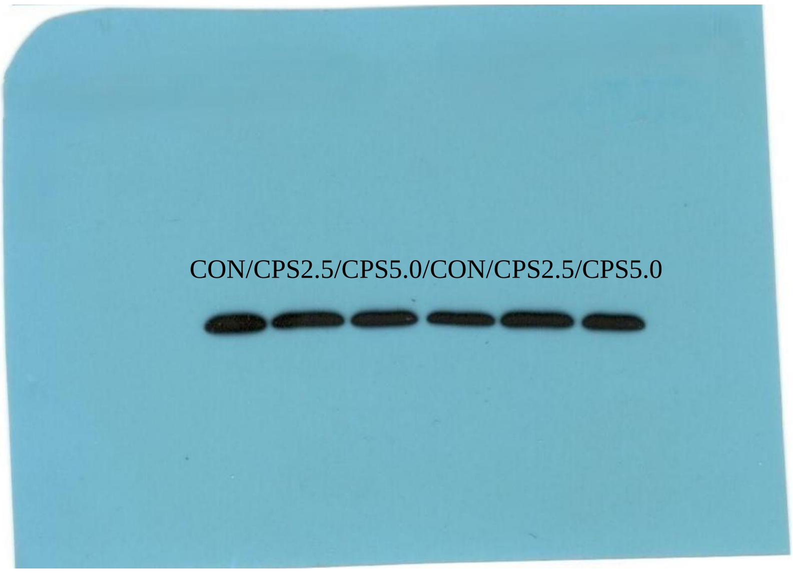

Supplement: Supplementary file 1 [file animals-15-00228-s001.zip › animals-3398044-WB Figures.pdf]
